# Turning the tide or surfing the wave? Responsible Research and Innovation, fundamental rights and neoliberal virtues

**DOI:** 10.1186/s40504-016-0038-2

**Published:** 2016-05-27

**Authors:** Simone Arnaldi, Guido Gorgoni

**Affiliations:** Interdepartmental Centre for Environmental, Ethical, Legal and Social Decisions on Emerging Technologies (CIGA), University of Padua, Padua, Italy; Department of Political Science, Law and International Studies, University of Padua, Padua, Italy

**Keywords:** Responsible Research and Innovation, Fundamental rights, Neoliberalism, Agency, Responsibility paradigms, Moral Entrepreneurship

## Abstract

The notion of Responsible Research and Innovation (RRI) has increasingly attracted attention in the academic literature. Up until now, however, the literature has focused on clarifying the principles for which research and innovation are responsible and on examining the conditions that account for managing them responsibly. Little attention has been reserved to exploring the political-economic context in which the notion of RRI has become progressively more prominent. This article tries to address this aspect and suggests some preliminary considerations on the connections between the specific understanding of responsibility in RRI and the framing of responsibility in what has been synthetically defined as ‘neoliberalism’. To do so, we try to illustrate how the idea of responsibility has evolved over time so that the specific characteristics of RRI can be better highlighted. These characteristics will then be discussed against the features of neoliberalism and its understanding of responsibility. Eventually, we reaffirm a view of RRI centred on fundamental rights as a possible point of departure between these two perspectives on responsibility.

The notion of Responsible Research and Innovation (RRI) has attracted growing attention. Up until now, the literature has focused on clarifying the principles for which research and innovation are responsible and on examining the conditions that account for managing them responsibly. Less attention has been reserved to exploring the political-economic context in which the notion of RRI is shaped and is gaining prominence as a discourse on and practice of governance. This article tries to address this aspect, by suggesting some preliminary considerations on the connections that can be established between the specific understanding of responsibility in RRI and the framing of responsibility in what has been synthetically defined as 'neoliberalism'.

The existence and features of these connections between neoliberalism and RRI are debated. Borrowing from David Guston’s comments on anticipatory governance, the inclusive approach RRI has towards governance is not meant “to acquiesce to neoliberal ideology that would focus on governance to the diminishment of government” (Guston 2014, 226). On the contrary, it is a response to the failures of markets “to manage innovation effectively for social good” (Mills 2013), rejecting market mechanisms as the sole source of “the normative dimension of what counts as an ‘improvement’” (Von Schomberg 2013, 54) and building collaborative mechanisms that are able to complement them or, in some cases, replace them altogether. However, this asserted distance from neoliberalism is contested, and critics maintain that RRI is instead suspectedly close to neoliberal governance, basically because it depoliticizes debate and deliberation (Pellizzoni 2015, van Oudheusden 2014). A different, but complementary, objection regards the risk that the values and assumptions of RRI can reproduce the dominant structural inequalities characterizing the world stage when this approach is cast against a global perspective (Macnaghten et al. 2014, 195).

To disentangle this contested relationship between neoliberalism and RRI, we closely examine their respective understandings of the link between the responsible agent and society, and of the nature and scope of responsible action. In our exploration, we first illustrate the features of neoliberalism and its understanding of responsibility. Subsequently, we examine the specific characteristics of RRI, framing this concept as part of evolving responsibility paradigms. We then compare their understanding of agency and responsibility, showing that there are, indeed, considerable conceptual similarities.

Despite this proximity, we maintain however that a clear distinction between the two can be drawn if, and only if, the “normative anchoring” of RRI on fundamental rights is maintained. Despite their deep similarities in structure, this emphasis on rights can discern RRI from a simple recast of a neoliberal approach to responsibility. Without denying the limits and the possible drawbacks of RRI in practice, we maintain that ‘taking RRI seriously’ implies complementing the aspiration to a collective engagement towards societally desirable and ethically acceptable outcomes of research and innovation with an explicit reference to fundamental rights as a normative bound for the direction of science, technology and innovation. In the EU context, this means specifically considering the Charter of Fundamental Rights of the European Union and the European Convention on Human Rights as essential pillars of RRI. Taken together, these two elements can make RRI utterly different from a neoliberal model of responsibility.

## Varieties of neoliberalism

We anticipated earlier in the introduction that the construction of the agent is the topic of our choice to critically discuss the contact points between the understanding of responsibility in RRI and in neoliberalism. However, before starting our comparative examination, it is useful to provide the premises of our argument, by introducing a few notes about ‘neoliberalism’ and its main features.

The notion of neoliberalism has long enjoyed a vast popularity in the public debate and in academic research alike. In both contexts, neoliberalism has often been used as a pejorative term to describe capitalistic and market-centred economic policies (Thorsen 2009). In this way, neoliberalism has frequently become a catchword, which has little analytical value. This essay tries to go beyond this characterization and to close this analytic gap. Our starting point is to acknowledge that the existence of a homogenous and coherent “neoliberal model” is questioned. Neoliberalism has been rather seen as a set of “actually existing” neoliberalisms, with a small’n’, which is characterized by a degree of conceptual openness and empirical diversification (Ong 2007) that makes it a “perplexingly amorphous political economic phenomena” (Peck 2004, 394). “In marked contrast to the strident clarity of neoliberal mantras, the real-world trajectories of neoliberalisation have been far more messy, following a proliferative array of pock-marked, crisis-strewn development paths“(Peck and Theodore 2012, 179).

This is also a consequence of the fact that neoliberal policies exist in “a more-than-neoliberal context. The context matters because it introduces difference, path dependency, and unevenness in terms of process and outcome: neoliberalisations in the plural” (Castree 2006, 3). In other words, concrete neoliberal arrangements are better understood as the hybrid results and flexible adaptation of market-oriented logics within changing regulatory landscapes (Blok 2012). As Collier and Ong affirmed (2005), neoliberalism co-exists with other political rationalities and its actual arrangements are the result of the interaction of global forms and situated political regimes and logics (see also the cited work of Ong 2007).

Such diversity has prompted the introduction of more variegated and processual notions like, for example, neoliberalisation (Brenner et al. 2010) or neoliberal globalization (Moore et al. 2011) as key notions to interpret the local variations and differentiated arrangements that characterize these “contemporary processes of market-oriented regulatory restructuring” (Brenner et al. 2010, 182).

Therefore, highlighting some broad tendencies in the neoliberal understanding of economics and politics beyond the local variations of political-economic arrangements and the institutional configurations in which notions and concepts are embodied, is surely a matter of simplification. However, some unity appears, at least at a very general level. On this, Harvey famously affirms the essence of neoliberalism is in the assumption that “human well-being can best be advanced by liberating individual entrepreneurial freedoms and skills within an institutional framework”, thus reorganizing the relationships between individuals, markets and States through “strong private property rights, free markets, and free trade” (Harvey 2005, 2).

This short definition describes the main features of this broad and variegated movement: the tendency to prefer markets over governments as policy instruments, the emphasis on property rights as the way to expand the market mechanisms across diverse and increasing areas of social life and nature, the aptitude to favour trade liberalization over protectionism, and the predilection of self-responsibility and entrepreneurship in managing economic issues and, as covered afterwards, life projects.

In this context, the market is seen as the central institution of society, and a benchmark for handling any type of social affair at any level. When compared to its classical antecedent, neoliberalism sees however that “market conditions are more explicitly constructed to optimise their beneficent role” (Levidow 2012, 161). The market is therefore considered as an artefact, an object of active construction, the result of “regulatory restructuring” and “market-based problem solving” strategies (Lave et al. 2010, 661). Because the market, market relations, especially competition, are seen as the result of this coordinated policy action, neoliberalism advocates the paradoxical “mobilization of state power in the contradictory extension and reproduction of market(−like) rule” (Birch 2006, 4; see also Lave et al. 2010).

In this context, individual property rights have a crucial role to enable the expansion of (free) market. Proprietisation and marketisation are seen by neoliberalism as the goals of a political programme. The expansion of “market relations into traditionally public arenas such as healthcare, education, and environmental management” (Lave et al. 2010, 661) is hence a matter of strategy and policy implementation, rather than in the nature of things. Hence, we have a prominent role of the rule of law, and the need to expand property rights over formerly unaffected social and biophysical sites (Levidow 2012).

## Constructing the entrepreneurial subject in (neo)liberal markets

Entrepreneurial subjects populate pervasive markets. As Ong notices, “[w]hile many consider neoliberalism broadly as global markets overwhelming countries, neoliberalism as a technique is fundamentally about the re-management of populations - about fostering self-actualizing or self-enterprising subjects” (Ong 2007, 5). In a Foucauldian fashion, neoliberalism can be seen as a “technology of governing”^1^. The key figure of neoliberalism is the entrepreneur, and the key social mechanism is competition, in a way that is consistent and, in a sense, underlies the neoliberal discourse and policy process aimed at promoting free market as an ultimate standard of economic and social organization. Indeed, such a deliberate transformation entails “a certain concept of what man is or should be” (Bárd 2010, 75), which accomplishes the extension of a sort of “capitalistic rule” to the self recasting “a theory of homo oeconomicus. [In neoliberalism, the] homo oeconomicus is a businessperson and a businessperson of him/herself […], being for oneself one’s own capital, being for oneself one’s own producer, being for oneself one’s own source of income”. Individual strategic calculation in the market is therefore directed to increasing this biological capital, to increasing return on the investment made on one’s own body” (Da Silva Medeiros 2006, 1). “[A]s Nikolas Rose notes, at the very moment when countless accounts of the passing and demise of the image of the self as stable, unified and autonomous emerge in philosophy and social theory, regulatory practices seek to govern individuals in a way which is more tied to their “selfhood” than ever before, and the ideas of identity and its cognates have acquired an increased salience in so many of the practices in which human beings engage” (Bárd 2010, 76).

The pervasiveness of the market logic in all domains of life makes entrepreneurship an ‘existential’ feature rather than only an economic one. For the “entrepreneurial, flexible self” (Bárd 2010, 75), “life is considered as a project the objective of which is to increase the person’s human capital. For that it is necessary to work actively on the self and to construct a ‘lifestyle’, with the ultimate goal of happiness” (Ferreira et al. 2012, 147). This active commitment is coupled with “a view of the self as autonomous, choosing, rational; someone who pursues its own life-plans according to its own values and priorities” in an increasingly turbulent world (Bárd 2010, 75). Risk taking and risk management are inherent to neoliberal subjects (Pellizzoni 2012). It is important, however, to notice that it is risk, or differently put, uncertainty and instability that create the very conditions of self-realization, i.e. of the individual definition and pursue of individual life (or, in a narrower fashion, economic) goals in an entrepreneurial way.

While this emphasis leads coherently to the clear prevalence of the individual on society, it does not mean that the latter is set to disappear. Rather, society is read through these invidividualised and responsibilised lenses. Lessenich has described this renewed view of society in terms of “neosociality”. Neosociality is “a new mode of political self justification of society vis-à-vis its individual members, constructing active subjects […] as *socialized selves* obliged not only to be responsible for themselves, but for society and its welfare as a whole [. G] governing people means relocating the promotion of the social into the individual, resubmitting it to the individual’s responsibility” (Lessenich 2010, 306). This entrepreneurial, pro-active self is the condition for the collective to seize the opportunities offered by the uncertain environment. Individuals are called to act prudently in order to ensure the benefit of society through their own individual actions, thus, the “common good” becomes the assumed maxim of responsible action (Lessenich 2010). As Maasen, Sutter and Duttweiler summarize the point, “[b]eing neosocial is thus tantamount to individuals that flexibly govern themselves and others by way of socially accepted means” (cited in Bárd 2010, 87).

By characterizing this push towards market economy in normative terms, neoliberalism describes therefore a teleological movement towards the Market (with a capital M). We maintain that this parallel movements describe two 'nested teleologies' (Arnaldi 2012), which apparently flourish in neoliberalism. The first is a 'systemic' one and it concerns the goal of a society fully shaped by the market as an institution encompassing all aspects of human life. The second one is nested in the former, and it is centred on the 'individual': a teleological view of the subject, whose agency, identity, and even self-fulfilment are tied to the individual capacity of strategic calculation, planning, and design of a 'life project' in a competitive (free) market environment. This normative characterization of the market supports and is, in turn, reinforced by an epistemic one. For neoliberalism, the market is not a mere economic phenomenon or a political goal. Markets are a goal worth pursuing because they are an epistemic phenomenon (Tyfield in Pellizzoni and Ylonen 2012, 6) and their function is primarily information processing. As such, they coordinate the individual and collective level. “On the one hand practices (e.g., patenting) must encourage and promote innovation and the capacity of commercial actors to enter (i.e., make) new markets so that the market can spread in new areas of life. On the other hand innovation has to be represented as a natural process in which 'fitness' (i.e., success) is (re)presented as a consequence of inherent and endogenous competitiveness thereby justifying and naturalising a specific set of practices” (Birch 2006: 2).

## Meanings of responsibility

After this short presentation of neoliberalism and the main features that are associated to this notion, it is now time to introduce the second key concept in this article: responsibility. More than a single concept, responsibility is “a syndrome of concepts” (Vincent 2011) variously interconnected. In order to achieve a richer and finer understanding of it many authors provide a list of different meanings associated with the idea of responsibility, which aims at better characterising the concept and at distinguishing its different theoretical and pragmatic dimensions. A classic effort, with an impact well beyond the legal field, was proposed by Hart (1968). More recent and refined taxonomies like, for instance, by Michael Davies (2012), Ibo van de Poel (2011), Nicole Vincent (2011), were significantly influenced by Hart’s work and developed his distinctions. For the purposes of the present article, these references are helpful for compiling a short list of meanings that can describe the different conceptual dimensions of responsibility.

1. *Capacity-responsibility* refers to the basic psychological/mental conditions for the ascription of responsibility (imputation).
2. *Causal-responsibility* results from being recognised as the origin (the “cause”) of a particular event.
3. *Liability-responsibility* implies the subjection to a sanction and/or compensation. This is often considered as being the primary sense of responsibility, particularly in law.
4. *Accountability-responsibility* indicates the obligation to justify (give reasons) for what one has done (or not).
5. *Role-responsibility* relates to the duties and tasks linked to a particular position.
6. *Virtue-responsibility* (Haydon 1978) implies a proactive engagement going beyond the compliance with an obligation mandated by the law. Whilst within the law clear reference is normally made to an ethics of (mere) compliance with the duty, the idea of virtue implies the reference to an “ethics of excellence” (Fuller 1969). Virtue responsibility, then, refers more to some valuable personal qualities of the agent. These qualities are related to the concept of agents’ responsiveness, emphasizing the idea of a disposition to listening and responding (Pellizzoni 2004, 557) rather than to the idea of an obligation to answering (either by giving an account or by liability, or both).

These meanings do not conclude the potential list of responsibility meanings^2^. These examples are, nevertheless, sufficient to capture two conceptually opposite semantic poles that are always present when responsibility is discussed: an active pole of responsibility assumption (undertaking responsibility, acting responsibly, mainly associated to the ideas of role, virtue,) and a passive pole of responsibility imputation (being held responsible, which is mainly associated to the ideas of causality, liability, accountability) (Bovens 1998, Arnaldi and Bianchi 2016) The passive to the active modalities of responsibility are not alternative but they indeed coexist (although not always without contrast).

The distinction between active and passive modes of responsibility entails another important aspect, namely time dimension, and under this aspect, we should distinguish between two temporal directions of responsibility: the retrospective and the prospective ones (Cane 2002). Retrospective responsibility (“historic responsibility”, Bovens 1998) is backward-looking, past-oriented, and essentially linked to the dimensions of imputation/ascription of responsibility and so to the dynamics of sanction, compensation or justification, which do belong to the passive idea of responsibility. Prospective Responsibility is forward-looking, future-oriented, and essentially linked to the dimensions of assumption and exercise of responsibility, connected with the ideas of performing roles and tasks both by complying with the duties associated to them, but also going beyond what is mandated and when the contents of duties and tasks cannot be established in advance. Prospective responsibility therefore emphasises the (pro)active dimension of responsibility that is captured by the idea of virtue-responsibility. In this sense, the idea of prospective responsibility appears to be more complex than that of a duty as it includes dimensions that are typically ethical (as capacity, virtue, moral obligation) as well.

In this active sense, responsibility implies actors’ “responsibilisation”. That is actors’ capacity of self-commitment towards some goals which are not mandated by rules (legal or of other sort). This is an eminently ethical feature, both at the individual and at the organisational level. “Responsibilization – namely expecting and assuming the reflexive moral capacities of various social actors – is the practical link that connects the ideal-typical scheme of governance to actual practices on the ground. Responsibility – in contrast to mere compliance with rules – presupposes one’s care for one’s duties and one’s un-coerced application of certain values as a root motivation for action” (Shamir 2008, 7). “Responsibilization is therefore fundamentally premised on the construction of moral agency as the necessary ontological condition for ensuring an entrepreneurial disposition in the case of individuals and socio-moral authority in the case of institutions”. (Ibidem).

Responsibilisation is therefore a governance strategy aiming at “predisposing actors to assume responsibility for their action” (Dorbeck-Jung and Shelley-Egan 2013) which, drawing from our discussion above, means predisposing actors to voluntarily assume *ex ante* responsibility for their action, overcoming the perspective of the pure rule-compliance. In this sense, responsibilisation strategies rely on the voluntary self-assignment of specific responsibilities, as well as the implementation of practical steps for their fulfillment^3^.

## The evolution of responsibility paradigms

As the idea of responsibility has evolved over time, different combinations of the active/passive and temporal dimensions discussed above have characterised its different framings and understandings. A short overview of these “paradigms of responsibility” can be useful to then place the RRI approach to responsibility in context and to highlight its peculiarities.

According to François Ewald (1993) we can distinguish three different paradigms of responsibility, corresponding to three different historical turns of the concept of responsibility, namely:

1. *the paradigm of fault*, corresponding to the traditional moral and legal idea of responsibility as linked to a faulty causation by the agent. Fault is the typical, or better the archetypical, form of responsibility (as it forms the core of the idea of responsibility). It is based on the moral obligation to respond and the subsequent subjection to liability (being subjected to an adverse treatment). This model of responsibility, which is central both in the legal and in the ethical field, is essentially retrospective as it is grounded on the judgement of a past action according to a set of given criteria and rules, and possibly the subsequent subjection to a sanction.
2. *the paradigm of risk*, typical in the industrial modernity, replaces a sanction with a compensation, thus overcoming the limits of the fault paradigm in addressing the increasingly relevant work accidents. The idea of risk, and the mechanisms of risk management through insurance, have the effect to disconnect responsibility from fault, making indemnisation independent from liability. This view on responsibility rests on the idea of social solidarity rather than individual responsibility. It separates the idea of responsibility from those of action, author and fault, and it links this notion to alternative references such as an event, a victim and risk (calculation), leading to the paradoxical de-responsibilisation of the agent, as their contribution to the production of the damage is irrelevant for the compensation mechanism to operate. Compensating victims against damages, without any reference to somebody’s fault, prevails on sanctioning those who are 'responsible', whose influence on a specific, adverse state of affairs becomes irrelevant according to this 'objective' logic of compensation. This shift in the understanding of responsibility had important, but ambiguous, legal and political consequences. On the one side, it greatly advanced the protection of accident victims (essentially workers, who did not need to demonstrate the fault of the entrepreneur in order to obtain economic compensation). On the other side, the mechanism of responsibility this paradigm entails is based on statistic calculations and it is completely disconnected from the role of the agent and their moral qualities, which were instead essential in the mechanism of fault. This model of responsibility is indeed prospective in that it aims at anticipating the occurrence of damages by means of risk calculation and management. This way, responsibility is oriented towards the future disclosing opportunities for action. However, at the same time, it remains linked to a retrospective logic in that it anticipates the occurrence of a damage but it does not imply an increased (ethical) responsibilisation of the actors concerned.
3. *the paradigm of safety* was introduced with the development, both in the ethical and in the legal thinking, of the idea of precaution. This development was consequent to the perceived inadequacy of the two previous paradigms to cope with the problems set by the evolution of science and technology, as they both presuppose either an identifiable author (fault) or some reliable data (risk) to assign responsibilities. The epistemic uncertainty affecting contemporary science and technology jeopardizes the possibility to calculate the probability of alternatives on which risk calculation is based. The precautionary principle stems from this new approach linking responsibility to uncertainty and focusing specifically on the preventive exercise of responsibility rather than on its subsequent ascription (be it via fault or risk management mechanisms). Precaution operates exactly where adequate guarantees against undesirable harmful consequences of scientific innovation cannot be provided by applying the general rules and standards of risk governance, so that the criteria for a responsible management of innovation have to be set case by case. It has been said that the precautionary principle delineates a sort of “law in situation” analogous to the ethical judgement (Papaux 2006) as the precautionary principle does not introduce new forms of liability nor new criteria of risk assessment, but rather focuses on actors’ responsibilisation, consequently promoting a prospective idea of responsibility rather than a retrospective one. Therefore, the dismissal of the risk-based understanding of responsibility comes with the re-assertion of the agent. This leads to sort of re-ethicising the idea of responsibility, but responsibilisation here is predominantly defined in negative terms: moral agency focuses on avoiding negative consequences, which becomes the only (or the most) morally acceptable objective of action.

Can we think of RRI as an emerging, distinct responsibility paradigm? A preliminary answer can be proposed by first examining the scope and meaning of the RRI notion. About this, despite some differences, the literature on RRI (von Schomberg 2013, Owen 2014, van den Hoven et al. 2013, Forsberg et al. 2015) shares a largely common understanding of responsibility and its dimensions.

1. *Responsibility is oriented to the future*: the specific approach of RRI does not aim only at sanctioning, compensating or preventing the negative consequences of innovation, like the fault, risk and safety paradigms did, respectively. Accordingly, RRI dismisses (the capacity of) prediction and control as the sole essential features of responsibility. It advocates instead a prospective idea of responsibility focused on its exercise, by way of steering innovation processes according to societal values and needs. In doing this, RRI takes the heritage of the Precautionary approach one step further and merges two typically separated perspectives on responsibility, namely the legal and the political one.
2. *Responsibility is proactive more than reactive*: responsibility is intended to be mainly a driving factor of the innovation process rather than a constraint. Therefore, the exercise of responsibility must extend beyond the boundaries of what is legally due/binding and must engage with the collective shaping of societally acceptable trajectories for research and innovation.
3. *Responsibility is a collective and participative process*: rather than being merely individual, responsibility is shared among different actors with different roles and powers along the innovation process. These actors are considered mutually responsible.
4. Different levels of Responsibility are strictly intertwined: RRI establishes a strong complementarity between different dimensions of responsibility, namely the political, legal, ethical, and economical ones. Indeed the pursue of responsible innovation rests on the voluntary adoption of standards which are not legally binding (ethical dimension of responsibility). These standards may become the normative references for RRI activities (political dimension of responsibility), so that our current “Grand Challenges” can be answered (social dimension of responsibility) respecting and promoting the EU Fundamental Rights (legal dimension of responsibility) at the same time^4^. Though this approach leaves space to contestation and disagreements, RRI encourages a logic of collaboration and shared commitments instead of an adversarial approach based on dispute settlement, be it at the judicial level or not.

These features seem to set RRI apart from the other responsibility paradigms we have briefly described above (see Table 1 for an inevitably simplified comparison). It does not mean that it replaces the other ones, but that it combines elements that already existed in a creative, and more comprehensive, fashion. Indeed, RRI can perhaps be considered as a new paradigm of responsibility that goes beyond the traditional emphasis on fault and punishment, risk and compensation, uncertainty and precaution. The priority is here on steering the innovation process from the inside towards societal goals rather than on coping with its (actual or anticipated) unwanted and unintended externalities.

**Table 1:** RRI and the evolution of responsibility paradigms

Paradigm	Criterion of ascription	Mean of realisation	Target	Dimension	Orientation in time	Responsibility dimensions	Regulating mechanism
Fault	Liability	Sanction	Negative outcomes	Individual	Retrospective	Liability-responsibility	Hard law
Risk	Damage	Compensation	Negative outcomes	Systemic	Prospective/Retrospective	Causality-responsibility	Hard law
Safety	Uncertainty	Precaution	Negative outcomes	Collective	Prospective/Anticipative	Capacity-responsibility	Hard law/Soft law
RRI	Responsiveness	Participation	Negative and positive outcomes	Collaborative	Prospective/Proactive	Virtue-responsibility	Self-regulation/Soft law/Hard Law

Adapted from Arnaldi, Gorgoni and Pariotti 2016

What interests more this article is, however, the way RRI constructs what we may call “the responsible subject”. The next section will address this aspect and, from that point of departure, we will then start our exploration of the existing similarities between RRI and the concept of responsibility in neoliberalism.

## RRI as an entrepreneurial model of responsibility

What are the features of the responsible subject in RRI? A comparison with the other paradigms can help to describe them.

As we have seen in the previous section, RRI fully restores the centrality of actors. In this, it is quite different from the risk paradigm and it is quite closer to the safety and fault ones. Yet, the responsible agent of RRI is significantly different from that of those paradigms. The RRI responsible agent is a proactive one. Agency does not matter only when retrospective ascription of responsibility and the possible corresponding sanctions are concerned. On the contrary, agency is predominantly prospective in RRI. This allows this emerging paradigm to overcome the objection challenging the paradigm of fault: uncertainty surrounding science and technology and its impacts make it impossible to establish the causal chains back to the (faulty) behaviour of agents that are necessary to determine liability. This prospective understanding of agency is, however, different from that of the safety paradigm as well. What distinguishes RRI from the precautionary attitude of the safety paradigm, is not their respective inner logic and their underlying epistemology (they both refer to decisions in a context of uncertainty), but rather their goals. The Precautionary Principle (PP) was meant as a safeguard against the undesirable outcomes of innovation activities, serving as a tool for correcting their path, either by inverting, diverting, or blocking them. RRI focuses on orienting science and technology along a (morally and socially) ‘right’ trajectory. Semantically, we might say that there is a shift from ‘precaution’ to ‘prudence’. In the paradigm of safety, the answer to uncertainty is (self-)restraint. In RRI, it is the prudent expansion of agency to determine and purposively steer research and innovation goals and activities. In terms of the various meanings of responsibility illustrated in the beginning of the article, we might say that responsibility in RRI is a matter of both virtue and capacity. The rescue of agency comes with the recuperation of ethics in responsibility, which was lost in the paradigm of risk. Such a recuperation is, as explained above, done on different grounds from the self-restraint that is advocated by the precautionary emphasis of the paradigm of safety and it implies the active commitment to achieve ethical goals in and by way of research and innovation. This re-ethicization of responsibility happens on grounds that are different from the paradigm of fault too, where it was basically the justification of liability and the consequent obligation to suffer an adverse treatment (sanction or blame). In other words, RRI is squarely placed in the perspective of responsibilisation, which is, as described above, a governance strategy aiming at predisposing actors to assume *ex ante* responsibility for their action (Dorbeck-Jung and Shelley-Egan 2013), overcoming a view of responsibility as liability or pure rule-compliance.

As responsibilisation strategies rely on the agent’s activity and spontaneous initiative, the voluntary self-assignment of specific responsibilities, and the implementation of practical steps for their fulfilment, it might be said that responsibility in RRI is essentially an entrepreneurial attitude and a disposition of the subject. It is not a coincidence that the responsible agent of RRI shares many of the characteristics that the literature (and the common people) associates to entrepreneurs: the willingness to prudently accept risks and to seize the corresponding opportunities, the capacity to mobilize the resources and actors to achieve her goals. Eventually, and probably most importantly, entrepreneurs have a propulsory role in social and organizational change, which is, as it is known, their crucial characteristic in the influential work of Joseph Schumpeter (Hamilton and Harper 1994, Pettigrew 1979, Hébert and Link 1989).

## A tale of two subjects: responsible agents in neoliberalism and RRI

The preceding sections have briefly described the main features of the two heterogeneous topics we are examining: Responsible Research and Innovation on the one hand, and neoliberalism on the other. At a first glance, a striking similarity concerns the insistence on a clearly entrepreneurial view of the responsible subject. The following paragraphs further examine this aspect, as it seems instructive to illustrate the similarities and differences between the ways in which RRI and neoliberalism define responsibility. We suggest the following dimensions as significant for this comparison:

1. *Temporal orientation*: responsibility is, in both cases, inherently future-oriented and proactive. The beacon of responsibility is the purposeful orientation of actions, as well as the prudent examination of their consequences.
2. *Direction of action*: this purposeful and prudential attitude is not confined to avoid possible negative, unintended consequences of one’s behaviour. On the contrary, agents have the moral duty to behave in a way to pursue desirable goals. As we have noticed above, the self-realisation of the entrepreneurial subject, and the removal of the obstacles to such achievement, is the signpost of the neoliberalism ‘teleological’ orientation. A similar ‘teleological’ orientation can be recognized in RRI and its emphasis on the finalization of research and innovation (and the related policies and practices) to the achievement of societal goals.
3. *Relation to uncertainty*: both RRI and neoliberalism assume uncertainty as opening opportunities for action rather than a motivation for adopting a merely precautionary stance. It is uncertainty that opens the possibility to purposefully pursue individual and collective goals. With uncertainty, comes opportunity. It is important to notice that we do not maintain that RRI excludes precaution, but the ambition to shape the trajectories of research and innovation in their initial stage is certainly predominant. This idea of powerful, yet prudential agency resounds in the neoliberal idea of reflexive, entrepreneurial agents maximizing the 'return on investment' of their actions and projects.

The dimensions we have listed and briefly commented above describe some important similarities between the idea of responsibility in RRI and in neoliberalism. The appraisal of both these two perspectives on responsibility requires, however, to be completed with an examination of what is seemingly different between the two. From this point of view, two aspects are particularly significant: the relation established between the responsible agent and society, the nature and scope of the purposes of (responsible) actions.^5^

With regard with the first dimension (the relation between the responsible agents and society they describe), the strong emphasis on individuals can deceive into reading an a-social (or even anti-social) logic in neoliberalism. However, a closer look can demonstrate this is not the case, as the discussion above has sought to clarify. On the contrary, neoliberalism has a distinct view on society and the links between individuals and the collective, which are commonly based on the “prudent management of self and others” that Lessenich (2011) associates to neosociality. Interestingly enough, the collective dimension of responsibility in RRI is the similar result of the interaction of “mutually responsive” societal actors (von Schomberg 2013, Owen et al. 2013), rather than, as occurring in what we have called the “paradigm of safety”, of objective conditions for and consequences of action that, in turn, determine a shared, collective, and equal responsibility (see, for instance, the logic underlying Jonas’s (1984) categorical imperative to maintain the possibility of human life on earth). What marks a difference between the two is how individual and collective responsibilities are coordinated. Neoliberalism is (neo)social insofar it considers common good as the consequence of the competitive interaction between calculating selves. As we have seen above, this seeming contradiction between competition and the common good is primarily solved by recurring to the market as the coordinating mechanism of such an interaction: the information processing capacity of the market links these two distinct levels in the most efficient way. On the contrary, RRI programmatically prefers collaboration over competition and conflict as a way for actors to coordinate responsibility: deliberation and involvement are the processes through which such coordination is sought. However, the apparent opposition between competition and cooperation is not a sufficient reason to consider RRI and neoliberal views of responsibility as irreconcilable. On the contrary, the literature has argued that mechanisms of participatory democracy can be included “in neoliberal forms of regulation” (Pellizzoni 2014, 215). With specific reference to RRI, we have argued elsewhere that RRI can be framed in the broader movement towards a “New Model of Governance” (Scott and Trubeck 2002). This New Governance model privileges participation and power sharing, integration of different levels of governance, diversity and decentralization, expansion of the space for stakeholders’ deliberation, flexibility and revisability, experimental and tentative nature over more traditional, nationally centralized, legally mandatory forms of regulation (Arnaldi, Gorgoni and Pariotti 2016, Pariotti 2011). This new regulatory regime has increasingly gained space in the governance of emerging science and technology, as an attempt to cope with the uncertain nature of their consequences and the wider and more heterogeneous constellations of actors that accompany their development. By preferring non-binding or voluntary regulatory instruments (Fredriksson et al. 2012, Skjærseth et al. 2006), this regulatory approach fully participates to the shift “from direct intervention (‘rowing’) to indirect intervention (‘steering’) in terms of enabling, motivating and pressing the regulated parties to regulate and to comply with self-regulation” (Dorbeck-Jung and Shelley-Egan 2013, 56). This is central to responsibilisation strategies and ultimately relies on the agent’s capacity to commit to some goals that are not mandated by rules with immediate, precise, direct, and uniformly binding effects, and with clearly delineated monitoring and enforcement authorities (Shaffer and Pollack 2012). In other words, it draws on the prudent, planful action of (relatively) unconstrained agents and on their self-disciplined behaviour. However, as we have explained above, these agent features are exactly those that one can recognize to the entrepreneurial neoliberal subject.

The second dimension we examine to draw a clear line separating the understanding of responsibility in RRI and neoliberalism is the *telos*, the purpose of responsible action. In discussing the neoliberal view of the subject, we noticed that their entrepreneurial efforts are aimed at self-realization. However, what self-realization means is quite undetermined. Self-realization is a goal in itself, it is self-justified as a continuous and progressive expansion of entrepreneurial agency, across societal domains and throughout individual life projects. At a systemic level, this individual search for self-realization is paralleled by the deliberate expansion of market as a regulatory mechanism of social relations, which is viewed as the best possible institutional context to make individuals tap their 'capital'. This open teleology of neoliberalism makes (metaphorical and actual) capital accumulation and return on investment as the purposes of action itself. As a consequence, responsibility boils down to individuals capacity to flexibly govern themselves and others so that the open-ended quest of self-realization can be sustained. Paradoxically, this teleological emphasis obscures ends to focus primarily on the means available to reach these unspecified goals.

While seemingly distant, we observe that the relation of RRI with the purposes of responsible action is not as straightforward as it may appear. In general terms (Forsberg et al. 2015), RRI advocates the reflexive and collective engagement with societal values, needs and goals, as a prerequisite to orient research and innovation practice and policy accordingly. From this generic point of view, the goals of research and innovation are disposable, in the sense that they are the result of public deliberation. It is not by chance that deliberation is another key dimension associated to RRI (e.g. Owen et al. 2013). We notice in passing that this is exactly the opposite of what happens in the paradigm of responsibility as safety. In the latter, safety is therefore objectively superordinate to other possible goals, because of the objectively acknowledged consequences of science and technology, as forcefully stated by Hans Jonas in his path-marking “principle of responsibility” (1984), and so it is precaution to other possible means. This is no longer true in RRI, where, as we have said, disposable purposes overcome objective conditions and the appeal to precautionary restraint becomes one option among the others. Though it is made in an effort to democratize science, technology and its responsible governance, the declared disposability of ends brings us back to the ambivalence of the link between (responsible) action and its purposes, which characterizes neoliberalism: intersubjective disposability not only concerns means, but also, and crucially, the ends of research and innovation. Once more, the concrete meaning of responsibility is left entirely to the stipulation of responsibilised agents, in the technical sense illustrated above.

### Distinguishing responsibility in neoliberalism and RRI: the role of fundamental rights

In the end, there are, therefore, considerable conceptual contiguities between the understanding of agency and responsibility in neoliberalism and in RRI. In our view, a clear point of departure of RRI from neoliberalism cannot be found either in the characteristics it assigns to the responsible agent, or in the way it frames the relation between individuals and collective responsibility. From our discussion above, the claim that their different conceptualization of the purposes of responsible action can set a clear distinction seems to be flawed too. In our view, the solution to this conundrum and the essential aspect that distinguishes RRI from a neoliberal understanding of responsibility is the notion of “normative anchoring”. In his widely cited essay on RRI, René von Schomberg defines RRI as “a transparent, interactive process by which societal actors and innovators become mutually responsive to each other with a view to the (ethical) acceptability, sustainability and societal desirability of the innovation process and its marketable products (in order to allow a proper embedding of scientific and technological advances in our society)” (Von Schomberg 2013, 39). In these works, ethical acceptability in the EU context “refers to a mandatory compliance with the fundamental values of the EU Charter on fundamental rights” (von Schomberg 2013, 40). Moreover, social desirability “captures the relevant, and more specific normative anchor points of the treaty on the European Union” (von Schomberg 2013, 40). The normative references contained in these legal documents therefore constitute the ‘building-blocks’ of a framework for the responsible governance of science and technology. This specific definition addresses the question of purposes in science, technology and innovation in a way that sets it apart from most of the RRI literature. Indeed, while a significant part of it refers to this aspect as the result of (normative) deliberations about S&T decisions (Owen et al. 2013), von Schomberg’s definition includes fundamental rights as the source of orientation for research and innovation (von Schomberg 2013). His definition explicitly grants a role to the legal dimension of RRI, complementing the mere reference to the normative orientation of innovation. The definitions of RRI that share this emphasis expressly link the ethical acceptability of research and innovation and the compliance with the EU Charter on fundamental rights, besides a general reference to safety as a paramount criterion for assessing technology and innovation (van den Hoven et al. 2013, 58). The societal needs innovation is expected to take account of are “expressed in the Treaty on European Union”, as sustainable development, equality, quality of life (van den Hoven et al. 2013, 58). This perspective explicitly links fundamental rights and societal needs in a comprehensive normative framework for the governance of science, technology and innovation.

Far from defining normative constraints top-down or limiting the scope and influence of public participation and deliberation, fundamental rights are neither abstract ideals or, worse, rhetorical pleas, nor rules with a definite, closed and compelling meaning that concerns solely the relationships between citizens on the one hand, and their governments or judicial courts on the other. Fundamental rights can, on the contrary, be thought of as claims that are justified by strong moral reasons and supported by legal norms, suitable to regulate both the (vertical) relations between the government and the citizens and, often, the (horizontal) relations among citizens themselves and, in general, among private actors (Arnaldi, Gorgoni and Pariotti 2016). The legal norms supporting the claims associated to these rights are structurally vague, because they have to apply to a number of cases that is as high as possible. This vagueness makes their content emerge also in a bottom-up fashion through the interaction among private actors and between them and the national and international public bodies, including judicial courts. In science, technology and innovation, this bottom-up process of meaning-making is particularly significant, as many private actors actively self-regulate and possess information and knowledge that is crucial to design and implement regulation. Therefore, it is possible to maintain that fundamental rights can be certainly one basic reference for any normative governance model and that, nevertheless, the development and implementation of such a model must necessarily come to terms with different values and with different interpretations of the rights themselves, consequently reflecting a diffuse and shared nature of responsibility. In sum, fundamental rights are not normative standards merely to be complied with and set in a top-down manner. Far from it, once listed, fundamental rights have to be filled with contents and have to be detailed with regard to specific domains, contexts, and cases. In this open-ended process of interpretation and application, societal values and norms can find (and usually do) a way of expression. So, the reference to fundamental rights does not involve any closure to public involvement and they can rather be seen as “a public normative practice” (Beitz 2009, 170).

Despite this open-ended nature, history has demonstrated nonetheless that such variations are cumulative and progressive, so that the rights progressively define their meaning and can gradually acquire a binding force (Ruggiu 2013, as the very consequence of the broad participation to this meaning-making process).

This twofold nature of fundamental rights, whose meaning is open yet determinate, therefore provides effective normative anchors to bound responsible action in research and innovation to defined ‘right’ ends. In this sense, fundamental rights are not simply constraints for innovation that aim to reduce or avoid its undesirable or negative consequences by warranting the respect of human health, dignity, privacy, etc. Rather, they also concern the shaping of policies, so that rights are not only respected and protected, but also promoted by way of proactive initiatives. This view of RRI centred on fundamental rights drops the indeterminacy of societal goals to anchor them to rights themselves. By doing this, it seems better placed to foster the consistency among different kinds of normative elements and to bound action to a stable normative orientation, yet open and flexible. By referring to rights, responsibilisation is filled with substance without losing space for public debate and participation. Rights do not deny agency and commitment. They require them instead. At the same time, they reduce the indeterminacy of purposes by anchoring entrepreneurial agents to specific normative standards and not to either the indefinite self-realization of neoliberalism or to a generic 'societal alignment' of research and innovation.

## Closing remarks: fundamental rights as the foundation of RRI

The idea of responsibility promoted by RRI has significant contact points with that promoted by neoliberalism and indeed RRI stems in an age marked by the seal of neoliberal approaches. Nevertheless, despite their deep similarities in structure, we do not conclude that RRI is simply a recast of a neoliberal approach to responsibility, even though their similarities suggest RRI policies and activities might be framed in the broader context of a neoliberal governance framework.

Without denying the limits and the possible drawbacks of RRI in practice, 'taking RRI seriously' means weighing the novel features it displays more consistently according its most ambitious definitions, and namely the idea of a collective engagement towards societally desirable and ethically acceptable outcomes of research and innovation.

In particular, we have highlighted two essential features which can set it apart from neoliberal approaches to responsibility, namely (1) its collective and cooperative nature and (2) its emphasis on the purposes of action. Unlike neoliberalism, which rests on markets and competition to coordinate responsibility at the individual and collective level, RRI demands a paradigm shift in that it requires a voluntaristic co-responsibility approach to ensure the convergence of differentiated responsibilities towards some common goals. This way, RRI is strongly defined in terms of cooperation and coordination (even in situations which do remain conflictual). This implies that RRI being constructed not only at the individual level, but also at the institutional level (broadly intended involving not only political institutions but also wider societal structures), by an interplay between system capacities and individual capabilities.

We tried to explain that, nonetheless, RRI normative commitment is not sufficient per se to decisively differentiate this approach from the neoliberal view of responsibility as a tension to self-realization. The demarcation comes when the normative orientation of research and innovation towards societal goals is filled with contents. We maintain that these contents are fundamental rights. In the European context, these are listed prominently in the European Convention on Human Rights (ECHR), in the EU Charter of Fundamental Rights, in the national constitutions, and the democratic standards which are closely interconnected with them. This puts RRI in a quite different setting compared to the neoliberal approach to responsibility, as individual entrepreneurship is not thought as a value per se. Instead, it becomes responsible insofar it is consistent with the values that are enshrined in fundamental rights and the related legal norms. It is this normative anchoring to fundamental rights that makes and can make RRI utterly different from a neoliberal model of responsibility. In this way, agency does not found limitation, but inspiration. Without anchors, the undirected disposability of purposes can create the risk that RRI can be realised in ways that de facto contradict its premises, thus becoming worthless rhetoric or an instruments for covering purposes other than its authentic promises.

## Footnotes

1 In her review of the literature, Popp Berman (2014) distinguishes three approaches to the analysis of neoliberalism: a governmentality approach, a marxist-oriented approach and a “roll-back neoliberalism”, which combines both. The biopolitical dimension that is implied by the governmentality approach is, however, crucial in distinguishing a more generic emphasis on economization and marketization from what can be defined as a “neoliberal” perspective. This distinctiveness is highlighted only to a limited extent in Berman analysis of US science policy.

2 Besides the Authors we have cited, see also, for instance, the notion of “care” (Pellizzoni 2004, Groves 2013) or “imputation” and “solidarity” (Ricoeur 2000).

3 In the regulatory context, a typical expression of this responsibilisation approach is self-regulation and its instruments, such as codes of conduct, guidelines, technical standards, reporting, audit, and so on (see Dorbeck-Jung and Shelley-Egan 2013, Arnaldi, Gorgoni and Pariotti 2016, Arnaldi 2014).

4 As we will see below, this latter and crucial aspect is considered only in part of the RRI literature.

5 In our view, this similar view of responsibility and agency has its roots in a similar view of nature as pliable and disposable. This peculiar plasticity of nature in neoliberal thinking and new technologies discourse has been explored in the recent literature (Pellizzoni 2012, 2015). While it is not possible to analyse this aspect in a detailed way, we suggest that the RRI discourse draws (at least partially) on a similar understanding.
